# A systematic review of the clinical and social epidemiological research among sex workers in Uganda

**DOI:** 10.1186/s12889-015-2553-0

**Published:** 2015-12-09

**Authors:** Katherine A. Muldoon

**Affiliations:** School of Population and Public Health, Faculty of Medicine, University of British Columbia, 2206 East Mall, Vancouver, BC V6T 1Z9 Canada; Ottawa Hospital Research Institute, University of Ottawa, 501 Smyth Road, Ottawa, ON K1H 8L6 Canada

**Keywords:** Sex work, Structural determinants, HIV/AIDS, Violence, Uganda

## Abstract

**Background:**

In response to the high burden of disease among sex workers and their position as a population heavily affected by the HIV epidemic, there has been a growing body of literature investigating the prevalence and risk factors associated with HIV risk among sex workers. To contextualize and summarize the existing research evidence base, a systematic review was conducted to synthesize the epidemiological literature on sex workers in Uganda.

**Methods:**

Database selection and search strategy development followed the Cochrane Collaboration’s standards for conducting systematic review searches. All studies that included sex workers as the primary research participants were included in the review. The search was then geographically restricted to the country of Uganda. Items were identified from 18 databases (grey and peer-review) on March 10–11, 2015.

**Results:**

A total of 484 articles were retrieved from the database search. After removal of duplicates, a total of 353 articles were screened for eligibility and 64 full-text articles were assessed. The final review included 24 studies with quantitative methodology conducted among sex workers in Uganda. The HIV prevalence among female sex workers ranged from 32.4–52.0 % and between 8.2–9.0 % had multiple HIV infections. Both multi-drug resistance to antiretroviral therapy (2.6 %) and antibiotics (83.1 %) were observed. Between 33.3–55.1 % reported inconsistent condom use in the past month. In the previous 6 months, over 80 % of sex workers experienced client-perpetrated violence and 18 % experienced intimate partner violence. Over 30 % had a history of extreme war-related trauma.

**Conclusions:**

There was limited information on socio-structural factors that affect sex workers’ commercial working environments in Uganda, including the role of policing and criminalization, as well as the prevalence and factors associated with violence. The majority of the existing evidence is based in Kampala, highlighting a need for information on sex work in other regions of Uganda. Additionally, there is limited information on features of the non-commercial components of sex workers’ lives as well as the services needed to reduce risks outside of the sex industry.

**Electronic supplementary material:**

The online version of this article (doi:10.1186/s12889-015-2553-0) contains supplementary material, which is available to authorized users.

## Background

The high prevalence of HIV among sex workers has been consistently documented from the beginning of the HIV epidemic [1]. Studies from the early 1980s began to document the high prevalence of HIV among sex workers within Uganda [2–4]. The Ugandan Government was quick to acknowledge the scale and severity of the HIV epidemic within the general population and began large scale campaigns to encourage condom use and reduce the number of sexual partners [5]. As a result, there is a large body of clinical research on HIV in Uganda, including many clinical trials, particularly among sero-discordant couples [6] and on the prevention of mother to child transmission [7], but much less research among key affected populations such as sex workers [8]. Country-wide prevalence estimates, based on available data, suggest that approximately one-third or 37.2 % (95 % CI: 34.2 %–40.2 %) of female sex workers are living with HIV in Uganda compared to 8.5 % among the general Ugandan female population of reproductive age [1]. The sustained levels of HIV suggest that there are larger structural barriers to health care and social and environmental factors that constrain sex workers’ choices and ability to reduce risk and exposure to HIV [9–11].

In response to the high burden of disease among sex workers and their position as a population heavily affected by the HIV epidemic, there has been a growing body of literature investigating the prevalence and risk factors associated with HIV risk among sex workers. In addition to the high burden of HIV/STIs among sex workers across sub-Saharan Africa [1, 11–14], there has also been a focus on the factors that influence sex workers’ ability to protect themselves, their clients, intimate partners, and families from risk [10]. Sex workers often have limited economic options and provide for many dependent children and family members, with limited access to education [11, 15–17].

In many countries in sub-Saharan Africa, sex work is criminalized and sex workers are often persecuted with little protection available from police or social services. In the Ugandan Penal Code, all aspects of sex work are considered illegal including the sale of sex, solicitation, communications for the purposes of sales, and third party entities such as clients, brothel owners, pimps/managers or those otherwise living off the earnings of prostitution [18]. Additionally, in 2014, the government of Uganda enacted the Anti-Pornography Act [19], previously known colloquially as the “Miniskirt Bill” [20]. In its original Bill form, pornography was described as a criminal offence and defined as “a) a person engaged in explicit sexual activities or conduct; b) exposing sexual parts of a person such as breasts, thighs, buttocks or genitalia; c) erotic behavior intended to cause sexual excitement; or d) any indecent act or behavior tending to corrupt morals.” The broad scope of the Bill was problematic and refined. When the Bill was enacted pornography was defined as “…any representation through publication, exhibition, cinematography, indecent show, information technology or by whatever means, of a person engaged in real or simulated explicit sexual activities or any representation of the sexual parts of a person for primarily sexual excitement”. The Anti-Pornography Act does not specifically use the terms prostitution or sex work; however, it further criminalizes the sex industry and provides sufficient legal infrastructure to arrest, detain, and harass sex workers.

Despite the development of the Anti-Pornography Act, the Ugandan government has made a commitment to improve access to HIV care and treatment for sex workers in the Ugandan National HIV Strategic Plan [21]. Qualitative research among sex workers in Uganda has shown that the criminalized status of sex work excuses extreme violence against sex workers, inhibits their ability to reduce HIV risk and negotiate condom use, and often displaces them to isolated and dangerous areas to avoid police harassment [22–25]. Despite the ongoing documentation of the increased HIV risk, to date there has been no acknowledgement from the Ugandan government that criminalizing sex work could undermine HIV prevention efforts.

Many studies have documented the extreme human rights abuses that sex workers face including homicide, physical and sexual violence, unlawful arrest or detention, and discrimination when accessing health services [14, 16, 26]. Sex workers will often forgo condom use because of fear of violence from clients, police or pimps/managers [10, 15, 16, 27]. Although there is limited epidemiological research, there is a growing body of qualitative research among Ugandan sex workers describing the common occurrence of violence from clients, including extreme acts of gang rape and physical assault [22, 23, 28–30]. Studies have documented that police can be both perpetrators and enablers of violence against sex workers [30]. To date, there is limited quantitative evidence on the burden of violence against sex workers in Uganda, an important and neglected area for investigation.

Reviews of the literature among sex workers in sub-Saharan Africa have documented a high unmet need for comprehensive health care services that include HIV treatment, prevention and care, sexual reproductive health, harm reduction, and psychosocial support [10, 31, 32]. Ensuring equitable and non-judgmental access to HIV care for sex workers in sub-Saharan Africa is a priority identified by sex work advocacy and community groups [24, 33] and endorsed by global governing bodies [34–36]. Even with examples of successful service provision, many remain small in scope and precariously funded, and few are able to address the structural drivers of risk including discrimination, violence, and lack of protections through criminalized law. Qualitative studies among sex workers in Uganda have cited discrimination from health care providers and difficulty accessing condoms as key barriers to care [24, 29]. Engaging sex workers in the continuum of HIV care is a necessary component of HIV prevention and treatment. Consequently, many sex workers may not know their HIV status, lack access to testing and treatment, and face stigma and discrimination from health care providers that inhibit retention in care [37, 38]. Specialized programming and effective interventions to increase condom use, access to HIV/STI testing and treatment, and access to primary and comprehensive care for sex workers are needed [39].

In response to the recognition of heightened risk of HIV/STI infection and transmission among sex workers, several studies have been conducted in Uganda. Uganda is an interesting case study where sex work is increasingly criminalized and yet sex workers are also acknowledged as a priority population for HIV prevention, treatment and care in national and global guidelines. Information on sex workers in Uganda spans national survey data, clinical trials and social analyses of sex worker health and safety. This systematic review was designed to be broad in scope and geographically restricted to Uganda to provide a country-level synthesis of sex workers across multiple disciplines. Results focus on the prevalence of HIV/STIs and factors that influence risk.

## Methods

### Search strategy

Prospero, the prospective registration platform for systematic reviews was searched on March 8, 2015 to determine if there was an existing systematic review registered or published on topics related to sex workers in Uganda [40]. None was found. A more sensitive search strategy was designed to retrieve studies from other electronic bibliographic databases. Database selection and search strategy development followed the Cochrane Collaboration’s current international Methodological standards for the conduct of new Cochrane Intervention Reviews (MECIR) standards for conducting systematic review searches [41]. Items were identified from the following databases on March 10–11, 2015: Cochrane Library via Wiley (Issue 3 of 12, 2015); Campbell Collaboration Library of Reviews; MEDLINE and PreMEDLINE via OVID (1946-present); PSYCINFO via OVID (1861-present); Sociological Abstracts via Proquest (1952-present); Dissertations and Theses via Proquest (1743-present); EconLit via Proquest (1982-present); IDEAS Economics and Finance Research via web (all years); British Library for Development Studies (BLDS) via web (all years); ISI-Web of Knowledge via Thompson; Web of Science Core Collection: Citation Indexes; Science Citation Index Expanded (SCI-EXPANDED) (1900-present); Social Sciences Citation Index (SSCI) --1900-present; Arts & Humanities Citation Index (A&HCI) --1975-present; Conference Proceedings Citation Index- Science (CPCI-S) --1990-present; Conference Proceedings Citation Index- Social Science & Humanities (CPCI-SSH) --1990-present; Health Evidence (database of public health systematic reviews) (all years); CAB Direct (CAB Abstracts & Global Health) (all years).

Searches were conducted for all years, in all languages. For maximum sensitivity, no study design filters were applied. Given the multidisciplinary nature of this topic, databases from the fields of medicine, health, social science, and economics, and sources of grey literature (i.e., IDEAS, BLDS) were selected. The search strategy was devised for the OVID Medline interface, tested using relevant, target articles, and then adapted for the other databases. The reference lists of papers selected for full text appraisal were scanned for additional potentially relevant material.

All references were imported into an EndNote Library and tagged with the name of the database. Duplicates were removed manually within EndNote, leaving a final total of 353 results (333 from the electronic databases and 20 through the grey literature and hand searching). The reporting of the search and selection results adhered to PRISMA guidelines (Preferred Reporting Items for Systematic Reviews and Meta-Analyses) [42]. Complete search strategies for all sources are available in Additional file 1.

The literature review was designed to identify any studies on sex work in Uganda. Sex work terms included: “sex work” or “sex workers” or prostitut* or brothel* or escort or “sex adj3 buy*” or “commercial adj3 sex*” or “sex adj3 industry.” Search terms to restrict articles to Uganda included: Uganda or Kampala or Kira or Mbarara or Mukono or Gulu or Nansana or Masaka or Kasese or Hoima or Lira or Mbale or Masindi or Njeru or Jinja or Entebbe or Arua or Wakiso or Busia or Iganga or Mpondwe or Kabale or Soroti or Mityana or Mubende.

### Inclusion/exclusion criteria

Two reviewers screened all full-text articles included in the search. Any discrepancies were resolved through discussion until consensus was reached. All studies that included sex workers as the primary research participants were included in the review. Sex work was defined as exchanging sex for money or other resources as a commercial activity. Sex workers could be self-identified as female, male or transgendered. The review was not restricted to any specific content area; however, only studies with quantitative methodology were included in the final review.

While both sex work and transactional sex are considered forms of economically motivated sex, commercial sex workers are considered a distinct key-affected population within the HIV epidemic and specific methodologies and standard questions have been developed to record their experiences. As a result studies that focused solely on transactional sex were excluded from the review. Transactional sex was defined as non-commercial or informal exchange of sex for resources. Studies where third-party participants reported on interactions with sex workers (e.g., military, truck drivers, community members, health service providers, etc.) were not included. This search was then restricted to research studies conducted in the country of Uganda.

### Data extraction and synthesis

The following data was extracted from the studies: source, setting, objective, design, sample size and characteristics, results including prevalence rates, and risk factors for various outcomes.

## Results

**Fig 1:**
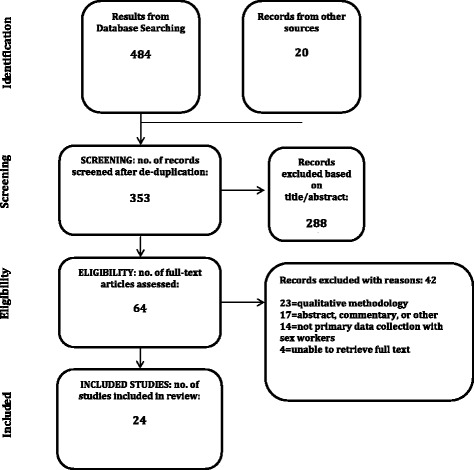
PRISMA flow chart: Study selection for systematic review of sex work in Uganda

Figure 1 provides the outlay for the study selection process. A total of 484 articles were retrieved from the database search; 20 were identified through the grey literature and hand searching. After removal of duplicates, a total of 353 articles were screened for eligibility based on title and abstract and 64 full-text articles were assessed.

The final review included 24 studies with quantitative methodology conducted among sex workers in Uganda (Table 1). The included studies were published between 1997 and 2015. There were 21 quantitative studies and three mixed method studies from eight original data sources. There were six studies from multi-national research collaborations. Three studies were conducted as part of a randomized controlled trial (RCT) investigating the clinical effectiveness of a vaginal microbicide gel to prevent HIV infection,^1^ with study sites in Uganda, South Africa, Benin, and India [8, 43, 44]. This trial was halted prematurely because there were higher rates of HIV acquisition among sex workers assigned to the intervention arm.

**Table 1:** Overview and characteristics of included studies

Source	Setting	Design	Objective	Sample size and characteristics	Results
Bukenya et al., 2013	Kampala	Longitudinal cohort: cross-sectional baseline analysis	To describe the prevalence and determinants of inconsistent condom use	*N* = 905 sexually active Ugandan female sex workers	40.2 % inconsistent condom use with paying clients in the last month. Increased risk: sex work not the sole source of income, sexual debut before 14 years, daily consumption of alcohol, fewer paying clients in last month, and currently pregnant. Decreased risk: currently married, higher number of sexual partners.
Erickson et al., 2015	Gulu, northern Uganda	Cross-sectional survey	To describe the prevalence and correlates of dual contraceptive use	*N* = 400 Ugandan female sex workers	45.0 % had ever used dual contraceptives.
					Increased odds of dual contraceptive use: older age, prior unintended pregnancy, HIV testing. Decreased odds of dual contraceptive use: rushing client negotiations because of police presence.
Francis et al., 2013	Multisite – Uganda (Kampala), Tanzania	Longitudinal cohort: cross-sectional analysis	To describe intervaginal cleansing among sex workers	*N* = 200 female sex workers, *n* = 100 Ugandan female sex workers	Among Ugandan sex workers: 81.8 % consistent condom use in past 3 months; 52.0 % HIV positive.
					Among Ugandan sex workers 100 % reported intravaginal cleansing ever; 4.5 cleansing acts per day; 80.3 % participants reported cleansing after half of their total sex acts. The frequency of cleansing was significant higher following sex, menstruation or vaginal discomfort.
Guédou et al., 2012	Multisite: Uganda (Kampala), South Africa, Benin, India	Double blind RCT: cross-sectional secondary analysis	To examine the association between prevalent intermediate vaginal flora (IVF), bacterial vaginosis (BV) and HIV infection among all sex workers screened for the RCT	*N* = 1367 female sex workers, *n* = 516 Ugandan female sex workers	Among total sample, 27.0 % HIV prevalence, 47.6 % BV prevalence, and 19.2 % IVF. BV and IVF were significantly associated with HIV.
					Among Ugandan sex workers, HIV prevalence 32.4 %, additional stratified analyses not available.
Guédou et al., 2013	Multisite: Uganda (Kampala), South Africa, Benin, India	Double blind RCT: longitudinal secondary analysis	To examine predictors of recurrent BV	*N* = 440 female sex workers with >1 episode of BV, *n* = 167 from Uganda	Among total sample, BV incident rate of 20.8 recurrences/100-person-months; Risk factors: vaginal cleansing increased risk; consistent condom use and vaginal candidiasis decreased risk.
					Among Ugandan sex workers, 7.9 recurrences/100 person-months. No additional stratified analyses available.
Matovu et al., 2012	Kampala	Cross-sectional survey	To assess sexual risk behaviours, condom use and STI infection among sex workers	*N* = 259 Ugandan female sex workers	55.1 % used condoms inconsistently in past month; 77.2 % self-reported STI in past 12 months; 86 % sought treatment 3 days after recognition of symptoms; consistent condom use was 72.1 % with causal partners, 40.8 % with regular partners, 6.3 % with spouses.
Morris et al., 2006	Multisite - Uganda/Kenya on the Mobassa-Kampala highway	Longitudinal study: diaries of sexual activity for 30 days	To exploring the effect of condom use among sex worker on the trans-Africa highway in contributing to HIV epidemic	*N* = 578 Ugandan/Kenyan female sex workers, *n* = 175 Ugandan	Total of 14072 sex acts, 77.7 % of sex acts used condoms; Modelling – using HIV prevalence of 30.0–50.0 % it was estimated there are 3200–4148 new HIV infections per year on the Mombasa-Kampala highway.
Morris et al., 2009	Multisite - Uganda/Kenya on the Mobassa-Kampala highway	Longitudinal study: diaries of sexual activity for 30 days	To describe sexual behaviour among sex workers on the Mombasa-Kampala highway, compare risk between Ugandan and Kenyan sex workers	*N* = 578 Ugandan/Kenyan female sex workers, *n* = 175 Ugandan	Compared to Ugandan sex workers, Kenyan sex workers had higher consistent condom use (79.2 % vs 73.9 %), more likely to use condom during sex act, higher condom use with regular clients.
					Compared to Ugandan bars, bars in Kenya were more likely to: have condom dispensers, (25.0 % vs 1.0 %); distribute or sell condoms, (73.9 % vs 47.6 %); and have more weekly condom distribution.
Muldoon et al., 2014	Gulu, northern Uganda	Cross-sectional survey	To examine the proportion of sex workers with a history of LRA abduction, access to post-abduction reintegration services and relative mental health	*N* = 400 Ugandan female sex workers, *n* = 129 with history of abduction	From a sample of 400 sex workers, 32.3 % had been abducted, 43.4 % had accessed a reintegration program. Mental health status was not significantly different between those who did and did not access a reintegration program. HIV prevalence 41.1 %.
Muldoon et al., 2015	Gulu, northern Uganda	Cross-sectional survey	To describe the prevalence and correlates of client violence, assess relationship between policing and client violence	*N* = 400 Uandan female sex workers	Most common forms of client violence: physical assault (58.7 %), rape (38.3 %), gang rape (15.8 %). HIV prevalence was 33.8 %. Inconsistent condom use was 84.0 %.
					Increased odds of client violence: rushing client negotiations because of police presence, servicing clients in a bar, inconsistent condom use with any client, working for a manager/pimp.
Pickering et al., 1997a	Fishing village in south-western Uganda	Longitudinal study: diaries of sexual activity for 6 months	To describe sexual mixing patterns inside and outside town	*N* = 26 Ugandan female sex workers	Women contributed 421 women-weeks; 15 were married and 42.0 % of sex partner were with commercial partner; 11 were single and 20.0 % of sex acts were with non-commercial partners; 90.0 % of contacts were from men resident in the village.
Pickering et al., 1997b	Trading town in south-western Uganda	Longitudinal study: diaries of sexual activity for 6 months	To describe sexual mixing patterns	*N* = 48 Ugandan female sex workers	Women contributed 789 women-weeks; average 5.8 clients per week; 10.0 % of clients were non-commercial; condom use was 99.0 % with commercial partners.
Pickering et al., 1997c	Trading town and fishing village in south-western Uganda	Longitudinal study: diaries of sexual activity for 6 months	To describe sexual mixing patterns	*N* = 81 Ugandan female sex workers	Women contributed 1280 women-weeks; 34 women from fishing villages and rural areas 90.0 % of sex acts with local men; 47 women from town contacts 87.0 % of sex partners were with with truck drivers or outside clients; 52.0 % were HIV positive, no significant difference by location.
Redd et al., 2014	Kampala	Longitudinal cohort: longitudinal clinical analysis	To determine the rates of HIV primary and super-infection among sex workers in Kampala	*N* = 85 HIV positive Ugandan female sex workers	The prevalence of HIV superinfection was 8.2 % (3.4/100 person-years) and was not significantly different from the rate of primary infection in the same population (3.7/100 person-years).
Schwitters et al., 2015	Kampala	Cross-sectional survey	To estimate the prevalence of client initiated violence in the previous 6 months among sex workers	*N* = 1467 Ugandan female sex workers	81.8 % had experienced at least one form of client-initiated violence in previous 6 months: 39.1 % physical abuse, 44.7 % verbal abuse, 49.1 % forced sex, 54.9 % not paid.
					Increased odds of violence: longer duration in sex work, more frequent client demand for unprotected sex, consumption of 5+ alcoholic drink, soliciting in outdoor spaces (e.g. streets, parks, parking lots etc.).
Ssemwanga et al., 2012a	Kampala	Longitudinal cohort: longitudinal clinical analysis	To identify prevalence of multiple HIV infections and associated features of partnership histories	*N* = 324 HIV-positive Ugandan female sex workers	9.0 % had multiple infections, sex workers working in same localities had phylogenetically similar viruses.
Ssemwanga et al., 2012b	Kampala	Longitudinal cohort: longitudinal clinical analysis	To classify HIV drug resistance among ART naïve women with new HIV diagnosis	*N* = 42 ART naïve Ugandan female sex workers with new HIV diagnosis	HIV drug resistance point prevalence estimate of 2.6 % (95 % confidence interval, 0.1 %–13.8 %).
Van Damme et al., 2008	Multisite: Uganda (Kampala), South Africa, Benin, India	Double blind RCT: primary analysis	To investigate efficacy of cellulose sulphate microbicide gel to reduce new HIV infection	*N* = 1398 HIV-negative female sex workers total; *N* = 303 Ugandan	Cellulose sulphate gel did not prevent HIV infection and may have increased the risk of HIV acquisition, hazard ratio 1.61 (0.9–3.0). Within Ugandan sub-group, sex workers reported 17–19 (med) sex partners, 19–21 (med) sex acts in previous 7 days. 97.5 % condom use per sex act. Additional stratified analyses not available.
Vandepitte et al., 2011	Kampala	Longitudinal cohort: cross-sectional baseline analysis	To examine baseline prevalence and risk factors of HIV and STIs	*N* = 1027 Ugandan female sex workers	HIV prevalence 37.0 %, gonorrhoea 13.0 %, chlamydia 8.9%, T. Vaginalis 17.1 %, BV 55.0 %, candida infection 11 %, HSV-2 antibodies 79.9%, active syphilis 10.0 %.
					Increased HIV risk: older age, widowed, lack of education, sex work as sole income, street-based sex work, not knowing HIV status, using alcohol and intravaginal cleansing with soap.
Vandepitte et al., 2012a	Kampala	Longitudinal cohort: cross-sectional baseline analysis	To assess the prevalence and determinants of *mycoplasma genitalium* (MG) among sex workers	*N* = 1025 endocervical swabs from Ugandan female sex workers	MG prevalence: 14.0 % - more prevalent in HIV+; less prevalent in older women, those who were pregnant but never gave birth. Associated with gonorrhoeae, candida, trichomonas vaginalis.
Vandepitte et al., 2012b	Kampala	Longitudinal cohort: cross-sectional baseline clinical analysis	To describe the symptoms and signs associated with MG among Ugandan sex workers	*N* = 1027 Ugandan female sex workers	MG prevalence 14.0 %, increased risk: dysuria and mucopurulent vaginal discharge.
Vandepitte et al., 2013	Kampala	Longitudinal cohort: longitudinal clinical analysis	To investigating the patterns of clearance and recurrence of untreated MG	*N* = 119 Ugandan female sex workers with MG	Overall clearance rate 25.7/100 person years; 55.0 % spontaneously cleared infection within 3 months, 83.0 % within 6 months, 93.0 % within 12 months. Infection recurred in 39.0 % of women.
Vandepitte et al., 2014a	Kampala	Longitudinal cohort: nested case control	To examining the temporal association between MG status prior to HIV infection	*N* = 168 Ugandan female sex workers, *n* = 42 cases, *n* = 126 controls	42 sex workers acquired HIV during the study, incident rate of 3.6/100 person years; Non-significant association between MG infection and HIV acquisition.
Vandepitte et al., 2014b	Kampala	Longitudinal cohort: longitudinal clinical analysis	To assess the prevalence and antimicrobial susceptibility patterns of gonorrhoea among sex workers in Kampala	*N* = 148 Ugandan female sex workers with diagnosis of gonorrhoea	83.1 % ciproflaxin resistance, 68.2 % penicillin resistance. 97.3 % tetracycline resistance.

Two studies were conducted among sex workers working along the Mombassa-Kampala corridor [45, 46]. The study collected information from 1007 bars at 47 truck stops, 8 truck stop sites were in Uganda, and 39 sites were in Kenya.

A total of 18 studies from 5 data sources were conducted solely within Uganda: 12 studies included sex workers from Kampala [8, 47–57]. Three studies were conducted among sex workers in trading towns near Lake Victoria in south-western Uganda [58–60]. Three studies were conducted among sex workers living in Gulu, northern Uganda [61–63]. All studies were among female sex workers; there were no studies with male or transgendered sex workers.

In the review process, 13 studies with qualitative methodology were identified [22–24, 28–30, 64–70]. The systematic review followed PRISMA guidelines and excluded qualitative studies and non-original research. However, the qualitative research gives critical context and voice to sex workers’ narratives and lived experiences. The 13 qualitative studies were reviewed and summarized separately in Additional file 2, and key narratives related to the research are summarized in the discussion of this review.

### Disease burden

Seven studies reported the HIV prevalence among Ugandan sex workers – three studies in Kampala, one study in south-western Uganda, and three in northern Uganda [43, 51, 59, 61–63, 71]. The HIV prevalence among female sex workers across the three regions ranged from 32.4 % to 52.0 %. Independent factors significantly associated with higher odds of HIV infection included older age (>25 years), being widowed, having less than primary education, sex work as sole income, street-based sex work, not knowing HIV status, using alcohol, and intravaginal cleansing with soap.

Eleven studies reported on the prevalence of STIs. The prevalence of bacterial vaginosis (BV) was between 47.6–55.0 % [43, 44, 50]. The prevalence of herpes simplex virus-2 (HSV-2) was 79.9 %, gonorrhoea was 13.0 %, trichomonas vaginalis was 17.1 %, candida infection was 10.9 %, active syphilis was 10.0 %, and chlamydia was 8.9 % [51]. Between 8.2–9.0 % of sex workers had multiple HIV infections [47, 48], with evidence suggesting that sex workers working in close proximity to each other had genetically similar viruses. Multidrug resistance to antiretroviral therapy was observed in 2.6 % of sex workers with HIV [49] and among a sub-sample of 148 sex workers with gonorrhoea, 83.1 % were resistant to ciproflaxin, 68.2 % were resistant to penicillin, and 97.3 % were resistant to tetracycline [52]. The prevalence of *Mycoplasma genitalium* (MG) infection was 14.0 %, and higher in HIV-positive women than in HIV-negative women [50, 53]. MG clearance was shown to be slower among sex workers living with HIV and those with lower CD4 cell counts [55]. MG infection was also shown to increase the risk of HIV acquisition [54]. Among a sample of 259 sex workers in Kampala, 76.5 % of sex workers self-reported an STI in the past 12 months [56].

## Condom use

Ten studies reported on patterns of condom use among sex workers with recall periods ranging from the previous week to the previous six months. Inconsistent condom use within the previous month ranged from 33.3–55.1 % [45, 51, 56, 72].

In analyses of Kenyan and Ugandan sex workers along the Mombasa-Kampala corridor, a combined estimate for both Kenyan and Ugandan sex workers revealed that condoms were used in 77.7 % of sex acts in the previous month [45]. Compared to Ugandan sex workers, Kenyan sex workers reported higher condom use per sex act (79.2 % vs. 73.9 %) and higher frequency of 100 % condom use (26.8 % vs. 18.9 %) [46].

Analyses within a large cohort of sex workers from Kampala (Good Health for Women Project) found that 40.2 % of sex workers reported inconsistent condom use in the previous month [72]. Factors significantly associated with inconsistent condom use included sex work not being the sole source of income (AOR = 1.54; 95 % CI: 1.13–2.09), sexual debut before 14 years (AOR = 1.46; 95 % CI: 1.09–1.96), daily consumption of alcohol (AOR = 1.90; 95 % CI: 1.26–2.88), and being currently pregnant (AOR = 2.11; 95 % CI: 1.25–3.57). The odds of inconsistent condom use were lower among those who were currently married (AOR = 0.36; 95 % CI: 0.18–0.73) and those with more than 10 clients per month (i.e. compared to those with less than 10 clients, those with 10–19 clients (AOR: 0.65, 95 % CI:0.44–0.96) and more than 20 clients (AOR:0.53, 95 % CI:0.39–0.75) had lower odds) [72]. In sub-analyses of sex workers with paying clients in the previous month, only 33.9 % reported consistent condom use [51], and among sex workers involved in a study that investigated intravaginal cleansing practices, 81.8 % reported using condoms consistently over the past 3 months [71].

Within the microbicide randomized control trial, Ugandan sex workers reported 97.5 % consistent condom use with clients in the last week [8]. A study among sex workers in Kampala found that 94.0 % of sex workers had reported using a condom at least once in the previous month; however, 55.1 % used condoms inconsistently. Condom use varied by partner type and was highest with casual partners (72.1 %), regular partners (40.8 %), and spouses (6.3 %) [56].

Studies among sex workers in a trading and fishing town in south-western Uganda reported between 94.0–99.0 % used condoms with commercial partners and 59.0 % of non-commercial partners in the previous six months [58, 59]. The authors cautioned that these findings were likely subject to social desirability bias. Two studies among sex workers in northern Uganda found that 84.0–85.0 % of sex workers reported inconsistent condom use with clients in the previous six months [62, 63].

One study included condom use as an independent variable in the regression modeling but did not include prevalence estimates, however client condom refusal (a proxy measure for inconsistent condom use) was significantly associated with increased the odds of physical, verbal, sexual violence, and economic deprivation in the previous 6 months [57].

### Access to health care and services

Six studies reported on access to health care and services [46, 51, 56, 61–63]. Three studies reported on access to sexual health care, including access to condoms and HIV/STI testing and treatment [46, 51, 56]. Between 62.3–97.8 % of sex workers had ever tested for HIV [51, 63]. Among sex workers in Kampala, 76.5 % (143/187) of sex workers self-reported an STI in the past 12 months and of those, 93.0 % (133/143) sought treatment and of those who sought treatment, 78.2 % (104/133) completed the treatment [56]. Between 55.0–64.4 % of sex workers report difficulty accessing condoms [62, 63]. An analysis of the sex work environment along the Mombasa-Kampala corridor found that compared to bars in Uganda, bars in Kenya were more likely to have condom dispensers (25.0 % vs. 1.0 %), distribute or sell condoms, (73.9 % vs. 47.6 %), and have more weekly condom distribution [46]. One analysis among sex workers with a history of abduction into the Lord’s Resistance Army found that 43.4 % had accessed a reintegration program for survivors of abduction [61].

Although no quantitative estimates were reported, three studies conducted in the fishing and trading towns in south-western Uganda cite that there was no health clinic or health services in the villages at the time of the research [58–60].

### Violence

Three studies reported on commercial forms of violence from clients against sex workers [57, 62, 63]. In the previous 6 months, between 49.0–82.2 % of sex workers had experienced at least one form of client-initiated violence [57, 62, 63], 39.1–58.7 % had experienced physical abuse, 44.7 % verbal abuse, 38.3–49.1 % forced sex acts, and 54.9 % had not been paid after sex [57, 62]. Rape from any partner in the last 6 months was 41.3 %. No studies reported on direct violence from police or the general public. Factors shown to significantly increase the odds of client violence were servicing clients in public spaces, inconsistent condom use with clients, working for a pimp/manager, rushing negotiation because of police presence [57, 62].

Two studies reported on non-commercial forms of violence against sex workers [57, 61]. The lifetime prevalence of rape was 49.0 % with the most common perpetrator from the last rape being an intimate partner (18.2 %), friend (8.2 %), authority figure (3.4 %), and family member (2.1 %). From a sample of 400 sex workers in northern Uganda, 32.3 % had been forcibly abducted into the Lord’s Resistance Army (LRA), considered a severe form of childhood trauma.

### Policing

Two studies reported on the prevalence of policing and found 37.3 % of sex workers had rushed negotiations because of police presence [62, 63], this was associated with 1.61 (95 % CI:1.03–2.52) increased odds of client violence. Between 27.8–29.6 % of sex workers reported that police presence affected where they solicited for clients [63]. Additionally, 26.5 % had been arrested [62].

## Discussion

The aim of this systematic review was to summarize the epidemiological research pertaining to sex workers living and working in Uganda. There were a total of 24 studies using quantitative methodology that were conducted over the past 18 years, reporting a high prevalence of HIV and other STIs among sex workers.

The largest source of information about sex workers in Uganda is from a cohort of over 1000 sex workers in Kampala [51]. It documented a high prevalence of HIV (37.0 %), and the presence of other STIs including gonorrhoaea, syphilis, and bacterial vaginosis. Both antiretroviral drug resistance and antibiotic drug resistance were identified within this cohort. The study identified a high prevalence of inconsistent condom use in the previous month (40.2 %). Early sexual debut and daily consumption of alcohol were statistically significantly associated with inconsistent condom use. Sub-analyses found a high prevalence of intravaginal cleansing practices [71]. This study collected information from 2008–2009, and is the main source information for several Government of Uganda national HIV reports [21, 73].

### Sex work environment

The epidemiological research about sex workers in Uganda is largely concentrated in Kampala. Twelve of the 18 studies conducted solely in Uganda included female sex workers from Kampala. Many investigations of sex workers in sub-Saharan Africa take place in urban centres and there are likely very large populations of sex workers working in Kampala. However, extending beyond urban capitals is an important area for future research with sex workers.

Five studies were conducted along the trans-Africa highway. Three studies were conducted in trading and fishing towns in south-western Uganda, which described complex sexual mixing patterns between commercial and non-commercial partners [58–60]. There was high reported condom use among commercial partners and low condom use with regular and non-commercial partners. Two qualitative studies among this population were identified through the search, which also reported that consistent condom use was more difficult with regular partners [64, 65]. Fishing communities surrounding Lake Victoria have high HIV prevalence rates and are considered a key population within the national HIV strategic plan in Uganda [21]. The role of sex work within fishing communities is an important area for ongoing investigation [74].

HIV research along trucking routes has been an important source of information regarding mobile populations and spread of HIV [75–79]. This review identified two studies conducted along the Mombasa-Kampala corridor [45, 46]. Within these studies, Kenyan and Ugandan sex workers reported a relatively high proportion of condom use (77.7 % of sex acts used condoms); however, mathematical modeling estimated that this level of inconsistent condom use could still contribute to 3200–4148 new HIV infections per year [45]. Due to high mobility, there are many challenges associated with HIV service provision for truck drivers and sex workers. One analysis that compared the sex work venues in Kenya to those in Uganda, found that the bars and lodges in Kenya were more likely to have condom dispensers, sell condoms, and have more weekly condom distributions [46]. Workplace based interventions that include condom distribution and peer-outreach at the venues along truck stops could be an important strategy for risk reduction and one that aligns with current recommendations for interventions that are grounded in community empowerment [31].

There were three analyses among sex workers from northern Uganda [61–63]. As northern Uganda has been heavily affected by war, it is a vulnerable environment where sex workers are likely working but are difficult to access. There have been some studies investigating the informal practice of transactional sex [66, 80, 81] and sex trafficking/slavery within the LRA [82–84]. These studies showcased a high volume of violence from clients, low access to reproductive health care, high prevalence of inconsistent condom use, and a high prevalence of severe war trauma.

### Commercial and non-commercial violence against sex workers

There were four studies documenting violence against sex workers in Uganda [57, 61–63]. One study among sex workers in Kampala found high levels of commercial violence from clients (<80 %) and non-commercial violence from intimate partners (18.2 %), friends (8.2 %), authority figures (3.4 %), and family members (2.1 %) [57]. Studies from northern Uganda reported that 49.0 % of sex workers had experienced client violence in the previous 6 months and the most common forms were physical assault (58.7 %), rape (38.3 %), gang rape (15.8 %) [62, 63]. A significant source of non-commercial violence was childhood trauma, where one study reported that 32.3 % of sex workers in northern Uganda had experienced extreme childhood trauma including a history of abduction into LRA [61].

Qualitative studies among sex workers in Uganda consistently documented that a serious threat to health and safety among sex workers was violence from clients, including physical and sexual assault, rape and gang rape [23, 24, 28, 29, 69]. In addition to being a serious human rights abuse, violence and fear of violence from clients is associated with increased risk of HIV infection and reduced condom use [85, 86]. The limited availability of epidemiological information on violence against sex workers and violence within the non-commercial aspects of their lives is an area that requires further investigation in Uganda. The integration of violence prevention into comprehensive HIV programming has been shown to be an effective and important strategy, both to reduce risk of HIV and to improve general health and safety for sex workers [10, 27, 31].

Two studies reported on the prevalence or experience of policing [62, 63], including police harassment, abuse, arrest, or displacement because of police presence. Policing was directly associated with increased odds of client violence. Additionally, 26.5 % had been arrested [62].

Documentation of the role of policing is an important measure for the consequences of the criminalization of sex work. This is particularly important in Uganda as the criminalization of the sex industry increases. Qualitative studies among sex workers in Uganda have documented the common occurrence of police harassment, including arbitrary arrest, degrading treatment, and physical and sexual violence [22–25, 30, 69]. Given the global consensus that the criminalization of sex work is one of the largest structural determinants increasing the risk of HIV among sex workers [87], ongoing documentation of the effects of policing and criminalization of sex work is needed. International policy bodies have specifically called for the removal of all criminal laws targeting sex work as necessary for both HIV prevention and the protection of human rights [26, 76, 88].

### Limitations

The limitations of this review must be acknowledged. First, as this review was designed to assess and synthesize research with quantitative results among sex workers in Uganda, qualitative studies were not included. Qualitative studies are a necessary component of understanding the contextual and region-specific challenges that sex workers face and are essential to design epidemiological studies that can quantify risks and factors that impede HIV prevention. In the review process, 13 studies with qualitative methodology were identified and a summary of the results has been included in Additional file 2 and the results are discussed within this manuscript. Second, it is likely that the included studies have limited generalizability to sex workers from other settings. The scope of the review was geographically restricted to Uganda with the goal to offer a complete synthesis of all quantitative studies in Uganda independent of the topic of the study. An important gap identified in this review is the lack of regional distribution; as a result, these studies primarily summarize research from Kampala and along the trans-Africa highway and are not generalizable to other regions of Uganda. Additionally, three studies originated from a clinical trial assessing the effectiveness of vaginal microbicides and self-report measures of condom-use were very high and potentially skewed because of reporting bias. Third, this review did not formally evaluate the quality of evidence in the primary research presented and the relative weight of evidence was not conducted. This does allow for a greater range of study designs and methodologies to be included and has allowed for the inclusion of studies that cover behavioural, social, and clinical epidemiology.

## Conclusion

This systematic review describes the extent of the epidemiological research on sex workers in Uganda and documents multiple risk factors that contribute to the heightened burden of HIV/STIs among sex workers including challenges with consistent condom use and access to care and services. There was limited information on socio-structural factors that affect sex workers’ commercial working environments in Uganda, including the role of policing and criminalization, as well as the prevalence and factors associated with violence from clients. The majority of the existing evidence comes from sex workers in Kampala, highlighting a need for information on sex work in other regions of Uganda. Additionally, there is limited information on features of the non-commercial components of sex workers’ lives as well as the services needed to reduce risks outside of the sex industry. Moving forward, evidence is needed to further inform programming that can reduce risks associated with HIV/STIs and violence. Specifically, community-based programming designed to reach sex workers and provide support and education to reduce harms is needed. Successful models of care delivery include sex worker/peer-led outreach programs to reach sex workers who may be more hidden and less likely to access services. Best practices for programming with sex workers includes involving sex workers in all aspects of research and programming. As research continues, a combination of both clinical and social aspects of sex workers lives is needed to ensure that potential for successful programming and policy are realized.

## Additional files

Additional file 1:
**Search Strategies and results summary.** (DOCX 40 kb) Additional file 2:
**Overview and characteristics of 13 qualitative studies of sex workers in Uganda.** (DOC 124 kb)

## Footnotes

1 CONRAD Sulfate randomized control trial http://www.isrctn.com/ISRCTN95638385
