# Supplementary material for: A systematic review of the clinical and social epidemiological research among sex workers in Uganda
Source: BMC Public Health. 2015 Dec 9;15:1226. doi: 10.1186/s12889-015-2553-0 (PMC4674940; doi:10.1186/s12889-015-2553-0)
Supplement: Additional file 1: — Search Strategies and results summary. (DOCX 40 kb) [file 12889_2015_2553_MOESM1_ESM.docx]

**Web-Appendix A: Search Strategies and results summary**

| Database and platform | Search date | # retrieved |
| --- | --- | --- |
| Cochrane Library via Wiley (Issue 3 of 12, 2015) | Mar 10, 2015 | 34 |
| Campbell Collaboration Library of Reviews via web | Mar 10, 2015 | 0 |
| MEDLINE and PreMEDLINE via OVID | Mar 10, 2015 | 108 |
| PSYCINFO via OVID | Mar 10, 2015 | 22 |
| Sociological Abstracts via Proquest | Mar 10, 2015 | 55 |
| Dissertations and Theses via Proquest | Mar 11, 2015 | 47 |
| IDEAS Economics and Finance Research via web | Mar 10, 2015 | 3 |
| BLDS British Library for Development Studies via web | Mar 10, 2015 | 10 |
| ProQuest Dissertations & Theses Global via Proquest | Mar 11, 2015 | 47 |
| ISI-Web of Knowledge via Thompson | Mar 11, 2015 | 118 |
| EconLit via Proquest | Mar 11, 2015 | 2 |
| Health Evidence via web | Mar 11, 2015 | 7 |
| CAB Abstracts & Global Health via CAB Direct | Mar 11, 2015 | 31 |
| Total | | 484 |
| Total screened after duplicates removed | | 351 |

**Ovid MEDLINE – Draft search strategy**

Database: Ovid MEDLINE(R) In-Process & Other Non-Indexed Citations and Ovid MEDLINE(R) <1946 to Present>

Search Strategy:

--------------------------------------------------------------------------------

1 exp Sex Workers/ (770)

2 prostitut*.ti,ab. (3418)

3 exp Prostitution/ (5223)

4 (sex adj3 industry).ti,ab. (261)

5 (sex adj3 tour*).ti,ab. (71)

6 brothel*.ti,ab. (376)

7 (anti adj3 prostitut*).ti,ab. (30)

8 escort.ti,ab. (599)

9 (sex adj3 buy*).ti,ab. (59)

10 (commercial adj3 sex*).ti,ab. (1619)

11 or/1-10 (9077)

12 exp Uganda/ (7605)

13 (northern adj province).ti,ab. (209)

14 (West adj Nile adj Province).ti,ab. (4)

15 (West adj Nile adj District).ti,ab. (41)

16 (West adj Nile adj sub-region).ti,ab. (1)

17 (Kampala or Kira or Mbarara or Mukono or Gulu or Nansana or Masaka or Kasese or Hoima or Lira or Mbale or Masindi or Njeru or Jinja or Entebbe or Arua or Wakiso or Busia or Iganga or Mpondwe or Kabale or Soroti or Mityana or Mubende).ti,ab. (2025)

18 Uganda.ti,ab. (8110)

19 or/12-18 (10634)

20 11 and 19 (108)

**Cochrane Library 2015 Issue 3**

**Search Name: Ugandan sex workers**

**Last Saved: 09/03/2015 20:19:00.344**

ID Search

#1 MeSH descriptor: [Sex Workers] explode all trees

#2 prostitut*

#3 MeSH descriptor: [Prostitution] explode all trees

#4 sex near/3 industry

#5 sex near/5 tourism

#6 brothel

#7 anti near/3 prostitution

#8 escort

#9 sex near/3 buyer

#10 commercial sex

#11 #1 or #2 or #3 or #4 or #5 or #6 or #7 or #8 or #9 or #10

#12 Uganda

#13 northern near province

#14 Kampala or Kira or Mbarara or Mukono or Gulu or Nansana or Masaka or Kasese or Hoima or Lira or Mbale or Masindi or Njeru or Jinja or Entebbe or Arua or Wakiso or Busia or Iganga or Mpondwe or Kabale or Soroti or Mityana or Mubende

#15 #12 or #13 or #14

#16 #11 and #15 - 33 items

**Campbell Collaboration library –
Volume 0 (2004) - Volume 11 (2015)**

[**http://www.campbellcollaboration.org/lib/?go=browse_issues**](http://www.campbellcollaboration.org/lib/?go=browse_issues)

Search strategy: hand searched, browsed all issues

**Database: PsycINFO <1806 to March Week 1 2015>**

**Search Strategy:**

--------------------------------------------------------------------------------

1 Prostitution/ (2562)

2 (sex adj2 work*).ti,ab. (2798)

3 (sex adj3 tour*).ti,ab. (107)

4 brothel*.ti,ab. (226)

5 (sex adj2 industry).tw. (323)

6 (anti adj3 prostitut*).ti,ab. (14)

7 escort.ti,ab. (118)

8 (call adj2 girl).tw. (14)

9 (anti adj3 prostitut*).ti,ab. (14)

10 (sex adj3 buy*).ti,ab. (64)

11 (commercial adj3 sex*).ti,ab. (764)

12 or/1-11 (4465)

13 Uganda.tw. (1835)

14 (northern adj province).ti,ab. (67)

15 (West adj Nile adj Province).ti,ab. (0)

16 (West adj Nile adj District).ti,ab. (0)

17 (West adj Nile adj sub-region).ti,ab. (0)

18 (Kampala or Kira or Mbarara or Mukono or Gulu or Nansana or Masaka or Kasese or Hoima or Lira or Mbale or Masindi or Njeru or Jinja or Entebbe or Arua or Wakiso or Busia or Iganga or Mpondwe or Kabale or Soroti or Mityana or Mubende).ti,ab. (318)

19 or/13-18 (1954)

20 12 and 19 (22)

**Sociological Abstracts via Proquest 1952-present**

uganda AND (sex work* OR prostitut*)
uganda AND SU.EXACT("Prostitution")

**ProQuest Dissertations & Theses Global**

(TI(Prostitution) OR TI(prostitute) OR TI(sex buyer) OR TI(sex work) OR TI(brothel) OR TI(sex industry) OR TI(anti-prostitution) OR TI(escot) OR TI(commercial sex) OR AB(Prostitution) OR AB(sex work*) OR AB(brothel) OR AB(sex industry) OR AB(anti-prostitution) OR AB(escot) OR AB(commercial sex)) AND (TI(Uganda)OR TI(Africa) OR AB(Uganda))

**Econlit via Proquest**

(TI(Uganda) OR AB(Uganda)) AND (prostitute OR prostitution OR sex worker OR sex work)

**IDEAS via** [**https://ideas.repec.org/search.html**](https://ideas.repec.org/search.html)

IDEAS uses the RePEc database. [RePEc](http://repec.org/) stands for "Research Papers in Economics" and is an internal name for a group working on the provision of electronic working papers.

Uganda + ("sex worker" | prostitute | "sex work" | prostitution)

Searched whole record, synonyms, all years

**BLDS British Library for Development Studies via** [**http://www.ids.ac.uk/publications/search/**](http://www.ids.ac.uk/publications/search/)

Repository of reports and publications in the area of global poverty and injustice

Search terms: Uganda, sex worker, prostitute, sex work, prostitution, brothel, sex industry

**Web of Science Core Collection: Citation Indexes**

Science Citation Index Expanded (SCI-EXPANDED) --1900-present

Social Sciences Citation Index (SSCI) --1900-present

Arts & Humanities Citation Index (A&HCI) --1975-present

Conference Proceedings Citation Index- Science (CPCI-S) --1990-present

Conference Proceedings Citation Index- Social Science & Humanities (CPCI-SSH) --1990-present

| # 3 | [**118**](http://apps.webofknowledge.com.proxy.bib.uottawa.ca/summary.do?product=WOS&doc=1&qid=6&SID=3BCN2TPHa8BIE5VRGOb&search_mode=AdvancedSearch&update_back2search_link_param=yes) | #1 AND #2  *Indexes=SCI-EXPANDED, SSCI, A&HCI, CPCI-S, CPCI-SSH Timespan=All years* |
| --- | --- | --- |
| # 2 | [**124,253**](http://apps.webofknowledge.com.proxy.bib.uottawa.ca/summary.do?product=WOS&doc=1&qid=3&SID=3BCN2TPHa8BIE5VRGOb&search_mode=AdvancedSearch&update_back2search_link_param=yes) | TI=(prostitute OR prostitution OR sex worker OR sex OR brothel OR sex industry)  *Indexes=SCI-EXPANDED, SSCI, A&HCI, CPCI-S, CPCI-SSH Timespan=All years* |
| # 1 | [**15,916**](http://apps.webofknowledge.com.proxy.bib.uottawa.ca/summary.do?product=WOS&doc=1&qid=4&SID=3BCN2TPHa8BIE5VRGOb&search_mode=AdvancedSearch&update_back2search_link_param=yes) | CU=Uganda OR TI=(Uganda or Kampala or Kira or Mbarara or Mukono or Gulu or Nansana or Masaka or Kasese or Hoima or Lira or Mbale or Masindi or Njeru or Jinja or Entebbe or Arua or Wakiso or Busia or Iganga or Mpondwe or Kabale or Soroti or Mityana or Mubende)  *Indexes=SCI-EXPANDED, SSCI, A&HCI, CPCI-S, CPCI-SSH Timespan=All years* |

**Health Evidence (database of public health systematic reviews)** <http://www.healthevidence.org/default.aspx>

Search term: Uganda

**CAB Direct (CAB Abstracts & Global Health)**
"sex workers" OR "prostitution" OR "prostitutes" AND "Uganda"

**Appendix 2. Overview and characteristics of 13 qualitative studies of sex workers in Uganda**

| **Source** | **Setting** | **Design** | **Objective** | **Sample size and characteristics** | **Results** |
| --- | --- | --- | --- | --- | --- |
| Gysels, 2001 | Trading town in south-western Uganda | Qualitative: semi-structured interviews | Examining the sexual cultural drivers and mediators among commercial sex workers at a roadside truck stop | N=12 Ugandan sex workers | Themes: Truck drivers (clients) use 'middle men' to connect them to sex workers. Sex workers reported preferring ‘middle men’ because they professionalize the transaction and help to ensure that the driver will pay and use condoms. |
| Gysels, 2002 | Trading town in south-western Uganda | Qualitative: life histories | Examining the sex work environment on trans-Africa highway in southwest Uganda | N=34 Ugandan sex workers | Themes: sex working environments included 1) sex work in back-street bars only; 2) waitress in bars who engage in sex work; 3) women who own their own bar and engage in sex work. Domestic violence with non-commercial partners was common (82.35%) and consistent condom use was more difficult to negotiate with regular partners |
| Ntozi, 2003 | Kampala, Kabale (western Uganda), and Lira (northern Uganda) | Qualitative: 3 focus groups with sex workers | Investigating sexual behaviour change among sex workers and other key affected populations (male/female adolescents, male/female street children, truck drivers, barmaids, sex workers | N=30 sex workers in 3 focus groups | Themes: Sex workers reported that poverty led them to sex work, inconsistent condom use is more common with regular partners than casual or one time. Client violence and condom refusal is common. HIV testing was harder to find in Lira, northern Uganda. |
| Zalwango, 2010 | Kampala | Qualitative: life histories, 7 repeated interviews over 6 months | Documenting pathways into sex work through marital separation, supporting children, and migrating to Kampala for a higher urban wage | N=96 Ugandan sex workers | Themes: The money women receive from selling sex and other work helped them to independently pay for their housing children’s school fees, and food for their family without receiving support from partners. In their narratives women portrayed themselves as mothers, wives, partners, friends and workers with self-esteem and the hope of improvement in their lives through their own efforts. |
| Schoemaker, 2012 | Kampala | Qualitative: ethnographic |  | N=68 Ugandan sex workers | Themes: Benefits of sex work included higher income, independent working schedule. Risks include dangerous work place, violent clients (physical and sexual violence - including gang rape), police be physically and sexually aggressive. Social discrimination from public, families, and service providers. Poverty drives unprotected sex, more urgent to get money to support then family than to worry about HIV |
| Mbonye, 2012 | Kampala | Qualitative: life histories | Investigate sex workers gender relations from childhood to adult life and how it led them to sex work | N=58 Ugandan sex workers | Themes: Many sex workers experienced childhood adversity - neglect, abuse from parents and teachers. Early unwanted pregnancy led many to leave school. Needing to earn money for childcare was main reason for starting sex work. Violence from clients and police was common |
| Mbonye, 2013 | Kampala | Qualitative: 3 in-depth interviews per participant | Exploring key risk factors associated with different sex work environments. | N=58 Ugandan sex workers | Themes: Common sex work solicitation venues included street/roadsides, bars and nightclubs. Common service venues included lodges, bars, dark alleyways or parking lots. Outdoor locations were more dangerous - increased violence, police harassment, stigma from public |
| Scorgie, 2013 | Multisite: Uganda (Kampala), Kenya, South Africa, Zimbabwe | Qualitative: 55 in-depth interviews, 12 focus groups | Exploring the impact of violence and related human rights abuses on the lives of sex workers, and how they have responded to these conditions, as individuals and within small collectives. | N= 136 sex workers, female (n=106), male (n=26) and transgendered (n=4);  n=25 sex workers from Ugandan | Themes: Client violence is a common threat and includes physical and sexual violence and client condom refusal. Policing was common and included being harassed, beaten and/or sexually assaulted by police, legitimized through criminalization. Sex workers often needed to exchange sex to get help from authorities, including landlords and brothel owners. Collective organization is difficult with mobile populations |
| Scorgie, 2013 | Multisite: Uganda (Kampala), Kenya, South Africa, Zimbabwe | Qualitative: 55 in-depth interviews, 12 focus groups | Exploring sex workers experience of seeking care in public and private clinics, and how services can be improved. | N= 136 sex workers, female (n=106), male (n=26) and transgendered (n=4);  n=25 sex workers from Ugandan | Key unmet health needs included diagnosis and treatment for sexually transmitted infections and insufficient access to condoms and lubricant. Denial of treatment for injuries following physical assault or rape and general hostility from public-sector providers was common. When possible, sex workers attended private services, citing higher quality and respect for dignity and confidentiality. Participants called for the decriminalization of sex work to help reduce stigmatisation, particularly to transgender and male sex workers. Alongside law reform, sex worker advocated for peer-led outreach. |
| Nyanzi, 2013 | Kampala and IDP camps in northern Uganda | Qualitative: ethnographic, participant observation, repeat in-depth interviews, focus group, policy review, media analysis | To explore sexual minority and sex work experiences within the HIV response among refugees in Uganda | N=54 male, female, transgendered and queer refugees involved in sex work | Themes: Violence from clients was very common, including homophobic rape, gang rape, and being drugged. Policing was an extra concern among refugees because of statelessness. Poverty was driving unprotected sex. Criminalization of sex work and homosexuality limiting access to care from all sources (UN, government, NGO) |
| Mbonye, 2014 | Kampala | Qualitative: 3 in-depth interviews per participant | Assess the magnitude, driving factors and consequences associated with alcohol consumption among sex workers | N=40 Ugandan sex workers | Themes: Many began drinking as an emotional coping strategy and to gain courage to engage in sex work. Consquences of alcohol use included difficulty negotiating condom use, clients buying sex workers drinks in place of paying for sex, and intoxicated clients were more violent |
| Marlow, 2014 | Kampala | Qualitative: in-depth interviews | Investigating sex workers experiences with post-abortion care | N=9 Ugandan sex workers who just had an abortion | Themes: Most common reasons for abortion included: not knowing father, consequence of rape, could not afford another child. Stigma against sex workers and the stigma of having an abortion was a dual barrier to care |
| Lees, 2014 | Multisite: Uganda (Kampala)/Tanzania | Qualitative: semi-structured interviews | Investigating the drivers and motivations for intervaginal practices | N= 176 sex workers,  n=96 from Uganda | Themes: intervaginal practices (douching, cleansing with soap/water, inserting herbs) were motivated by overlapping concerns with hygiene, morality, sexual pleasure, fertility, relationship security, and economic security |
