# Supplementary material for: A systematic review of the clinical and social epidemiological research among sex workers in Uganda
Source: BMC Public Health. 2015 Dec 9;15:1226. doi: 10.1186/s12889-015-2553-0 (PMC4674940; doi:10.1186/s12889-015-2553-0)
Supplement: Additional file 2: — Overview and characteristics of 13 qualitative studies of sex workers in Uganda. (DOC 124 kb) [file 12889_2015_2553_MOESM2_ESM.doc]

Table 1. Overview and characteristics of included studies

| **Source** | **Setting** | **Design** | **Objective** | **Sample size and characteristics** | **Results** |
| --- | --- | --- | --- | --- | --- |
| Bukenya et al, 2013 | Kampala | Longitudinal Cohort: cross-sectional baseline analysis | To describe the prevalence and determinants of inconsistent condom use | N=905 sexually active Ugandan female sex workers | 40% inconsistent condom use with paying clients in the last month. Increased risk: sex work not the sole source of income, sexual debut before 14 years, daily consumption of alcohol, fewer paying clients in last month, and currently pregnant. Decreased risk: currently married, higher number of sexual partners |
| Erickson et al, 2015 | Gulu, northern Uganda | Cross-sectional survey | To describe the prevalence and correlates of dual contraceptive use | N=400 Ugandan female sex workers | 45.0% had ever used dual contraceptives.  Increased odds of dual contraceptive use: older age, prior unintended pregnancy, HIV testing. Decreased odds of dual contraceptive use: rushing client negotiations because of police presence |
| Francis et al, 2013 | Multisite – Uganda (Kampala), Tanzania | Longitudinal Cohort: cross-sectional analysis | To describe intervaginal cleansing among sex workers | N=200 female sex workers,  n=100 Ugandan female sex workers | Among Ugandan sex workers: 81.8% consistent condom use in past 3 months; 52.0% HIV positive;  100% reported intravaginal cleansing ever; 4.5 cleansing acts per day; 80.3% participants reported cleansing after half of their total sex acts. The frequency of cleansing was significant higher following sex, menstruation or vaginal discomfort |
| Guédou et al, 2012 | Multisite: Uganda (Kampala), South Africa, Benin, India | Double blind RCT: cross-sectional secondary analysis | To examine the association between prevalent vaginal flora abnormalities, BV and HIV infection among all sex workers screened for the RCT | N=1367 female sex workers, n=516 Ugandan female sex workers | Among total sample, 27.0% HIV prevalence, 47.6% BV prevalence, and 19.2% IVF. BV and IVF were significantly associated with HIV.  Sub-analyses: Among Ugandan sex workers, HIV prevalence 32.4%, additional stratified analyses not available. |
| Guédou et al, 2013 | Multisite: Uganda (Kampala), South Africa, Benin, India | Double blind RCT: longitudinal secondary analysis | To examine predictors of recurrent bacterial vaginosis | N=440 female sex workers with >1 episode of BV, n=167 from Uganda | Among total sample, BV incident rate of 20.8 recurrences/100-person-months; Risk factors: vaginal cleansing increased risk; consistent condom use and vaginal candidiasis decreased risk  Sub-analyses: Among Ugandan sex workers, 7.9 recurrences/100 person-months. No additional stratified analyses available. |
| Matovu et al, 2012 | Kampala | Cross-sectional survey | To assess sexual risk behaviours, condom use and STI infection among sex workers | N=259 Ugandan female sex workers | 55.1% used condoms inconsistently in past month; 77.2% self-reported STI in past 12 months; 86% sought treatment 3 days after recognition of symptoms; Consistent condom use was 72.1% with causal partners, 40.8% with regular partners, 6.3% with spouses |
| Morris et al, 2006 | Multisite - Uganda/Kenya on the Mobassa-Kampala highway | Longitudinal study: diaries of sexual activity for 30 days | To exploring the effect of condom use among sex worker on the trans-Africa highway in contributing to HIV epidemic | N=578 Ugandan/Kenyan female sex workers, n=175 Ugandan | Total of 14072 sex acts, 77.7% of sex acts used condoms; Modelling – using HIV prevalence of 30-50% it was estimated there are 3200-4148 new HIV infections per year on the Mombasa-Kampala highway |
| Morris et al, 2009 | Multisite - Uganda/Kenya on the Mobassa-Kampala highway | Longitudinal study: diaries of sexual activity for 30 days | To describe sexual behaviour among sex workers on the Mombasa-Kampala highway, compare risk between Ugandan and Kenyan sex workers | N=578 Ugandan/Kenyan female sex workers, n=175 Ugandan | Compared to Ugandan sex workers, Kenyan sex workers had higher consistent condom use (79% vs 74%), more likely to use condom during sex act, higher condom use with regular clients,  Compared to Ugandan bars, bars in Kenya were more likely to: have condom dispensers, (25% vs 1%); distribute or sell condoms, (73.9% vs 47.6%); and have more weekly condom distribution. |
| Muldoon et al, 2014 | Gulu, northern Uganda | Cross-sectional survey | To examine the proportion of sex workers with a history of LRA abduction, access to post-abduction reintegration services and relative mental health | N=400 Ugandan female sex workers, n=129 with history of abduction | From a sample of 400 sex workers, 32.25% had been abducted, 53 (43.4%) had accessed a reintegration program. Mental health status was not significantly different between those who did and did not access a reintegration program. HIV prevalence 53 (41.09%) |
| Muldoon et al, 2015 | Gulu, northern Uganda | Cross-sectional survey | To describe the prevalence and correlates of client violence, assess relationship between policing and client violence | N=400 Uandan female sex workers | Most common forms of client violence: physical assault (58.7%), rape (38.3%), gang rape (15.8%). HIV prevalence 33.8%. Inconsistent condom use 84.0%  Increased odds of client violence: rushing client negotiations because of police presence, servicing clients in a bar, inconsistent condom use with any client, working for a manager/pimp. |
| Pickering et al, 1997a | Fishing village in south-western Uganda | Longitudinal study: diaries of sexual activity for 6 months | To describe sexual mixing patterns inside and outside town | N=26 Ugandan female sex workers | Women contributed 421 women-weeks; 15 were married and 42% of sex partner were with commercial partner; 11 were single and 20% of sex acts were with non-commercial partners; 90% of contacts were from men resident in the village. |
| Pickering et al, 1997b | Trading town in south-western Uganda | Longitudinal study: diaries of sexual activity for 6 months | To describe sexual mixing patterns | N=48 Ugandan female sex workers | Women contributed 789 women-weeks; average 5.8 clients per week; 10% of clients were non-commercial; condom use was 99% with commercial partners |
| Pickering et al, 1997c | Trading town and fishing village in south-western Uganda | Longitudinal study: diaries of sexual activity for 6 months | To describe sexual mixing patterns | N=81 Ugandan female sex workers | Women contributed 1280 women-weeks; 34 women from fishing villages and rural areas 90% of sex acts with local men; 47 women from town contacts 87% of sex partners were with with truck drivers or outside clients; 52% were HIV positive, no significant difference by location |
| Redd et al, 2014 | Kampala | Longitudinal Cohort: longitudinal clinical analysis | To determine the rates of HIV primary and super-infection among sex workers in Kampala | N=85 HIV positive Ugandan female sex workers | The prevalence of HIV superinfection was 8.2% (3.4/100 person-years) and was not significantly different from the rate of primary infection in the same population (3.7/100 person-years) |
| Schwitters et al, 2015 | Kampala | Cross-sectional survey | To estimate the prevalence of client initiated violence in the previous 6 months among sex workers | N=1467 Ugandan female sex workers | 81.8% had experienced at least one form of client-initiated violence in previous 6 months: 39.1% physical abuse, 45% verbal abuse, 50% forced sex, 56% not paid.  Increased odds of violence: longer duration in sex work, more frequent client demand for unprotected sex, consumption of 5+ alcoholic drink, soliciting in outdoor spaces (e.g. streets, parks, parking lots etc) |
| Ssemwanga et al, 2012a | Kampala | Longitudinal Cohort: longitudinal clinical analysis | To identify prevalence of multiple HIV infections and associated features of partnership histories | N=324 HIV-positive Ugandan female sex workers | 9% had multiple infections, sex workers working in same localities had phylogenetically similar viruses |
| Ssemwanga et al, 2012b | Kampala | Longitudinal Cohort: longitudinal clinical analysis | To classify HIV drug resistance among ART naïve women with new HIV diagnosis | N=42 ART naïve Ugandan female sex workers with new HIV diagnosis | HIV drug resistance point prevalence estimate of 2.6% (95% confidence interval, 0.07%-13.8%) |
| Van Damme et al, 2008 | Multisite: Uganda (Kampala), South Africa, Benin, India | Double blind RCT: primary analysis | To investigate efficacy of cellulose sulphate microbicide gel to reduce new HIV infection | N=1398 HIV-negative female sex workers total; N=303 Ugandan | Cellulose sulphate gel did not prevent HIV infection and may have increased the risk of HIV acquisition, hazard ratio 1.61 (0.86-3.01). Within Ugandan sub-group, sex workers reported 17-19 (med) sex partners, 19-21 (med) sex acts in previous 7 days. 97.5% condom use per sex act. Additional stratified analyses not available |
| Vandepitte et al, 2011 | Kampala | Longitudinal Cohort: cross-sectional baseline analysis | To examine baseline prevalence and risk factors of HIV and STIs | N=1027 Ugandan female sex workers | HIV prevalence 37%, gonorrhoea 13%, trachomatis 9%, T. Vaginalis 17%, BV: 56%, candida infection 11%, HSV-2 antibodies 80%, active syphilis 10%.  Increased HIV risk: older age, widowed, lack of education, sex work as sole income, street-based sex work, not knowing HIV status, using alcohol and intravaginal cleansing with soap. |
| Vandepitte et al, 2012a | Kampala | Longitudinal Cohort: cross-sectional baseline analysis | To assess the prevalence and determinants of mycoplasma genitalium (MG) among sex workers | N=1025 endocervical swabs from Ugandan female sex workers | MG prevalence: 14% - more prevalent in HIV+; less prevalent in older women, those who were pregnant but never gave birth. Associated with gonorrhoeae, candida, trichomonas vaginalis |
| Vandepitte et al, 2012b | Kampala | Longitudinal Cohort: cross-sectional baseline clinical analysis | To describe the symptoms and signs associated with MG among Ugandan sex workers | N=1027 Ugandan female sex workers | MG prevalence 14%, increased risk: dysuria and mucopurulent vaginal discharge, |
| Vandepitte et al, 2013 | Kampala | Longitudinal Cohort: longitudinal clinical analysis | To investigating the patterns of clearance and recurrence of untreated MG | N=119 Ugandan female sex workers with MG | Overall clearance rate 25.7/100 person years; 55% spontaneously cleared infection within 3 months, 83% within 6 months, 93% within 12 months. Infection recurred in 39% of women. |
| Vandepitte et al, 2014a | Kampala | Longitudinal cohort: nested case control | To examining the temporal association between MG status prior to HIV infection | N=168 Ugandan female sex workers, n=42 cases, n=126 controls | 42 sex workers acquired HIV during the study, incident rate of 3.66/100 person years; Non-significant association between MG infection and HIV acquisition |
| Vandepitte et al, 2014b | Kampala | Longitudinal Cohort: longitudinal clinical analysis | To assess the prevalence and antimicrobial susceptibility patterns of gonorrhoea among sex workers in Kampala | N=148 Ugandan female sex workers with diagnosis of gonorrhoea | 83.1% ciproflaxin resistance, 68.2% penicillin resistance. 97.3% tetracycline resistance |

Web-Appendix B: Overview and characteristics of 13 qualitative studies of sex workers in Uganda

| **Source** | **Setting** | **Design** | **Objective** | **Sample size and characteristics** | **Results** |
| --- | --- | --- | --- | --- | --- |
| Gysels, 2001 | Trading town in south-western Uganda | Qualitative: semi-structured interviews | To examine the physical and social environment among commercial sex workers at a roadside truck stop | N=12 Ugandan female sex workers | Themes: Truck drivers (clients) use 'middle men' to find sex workers. Sex workers reported preferring ‘middle men’ because they professionalize the transaction and help to ensure that the driver will pay and use condoms. |
| Gysels, 2002 | Trading town in south-western Uganda | Qualitative: life histories | To examine the sex work environment on trans-Africa highway in southwest Uganda | N=34 Ugandan female sex workers | Themes: main sex work environments included working: 1) in back-street bars only; 2) as a bar waitress who also engages in sex work; 3) own a bar and also engage in sex work. Domestic violence with non-commercial partners was common (82.35%) and consistent condom use was more difficult to negotiate with regular partners. |
| Ntozi, 2003 | Kampala, Kabale (western Uganda), and Lira (northern Uganda) | Qualitative: 3 focus groups with sex workers | To investigate sexual behaviour among sex workers and other key affected populations (male/female adolescents, male/female street children, truck drivers, barmaids, sex workers) | N=30 Ugandan female sex workers in 3 focus groups | Themes: Poverty was the strongest determinant of entry into sex work. Inconsistent condom use is more common with regular partners compared to casual/one time clients. Client violence and condom refusal is common. HIV testing was harder to find in Lira, northern Uganda compared to Kampala and Kabale. |
| Zalwango, 2010 | Kampala | Qualitative: life histories, 7 repeated interviews over 6 months | To document pathways into sex work through marital separation, supporting children, and migrating to Kampala for a higher urban wage | N=96 Ugandan female sex workers | Themes: Sex work was an independent source of income to support their families (e.g. pay for housing, children’s school fees, food etc). Beyond sex work, women described themselves as mothers, wives, partners, and friends. They aimed to improve their lives through their own agency. |
| Schoemaker, 2012 | Kampala | Qualitative: ethnographic | To explore perceptions of HIV risk and the risks and benefits of being a sex worker | N=68 Ugandan female sex workers | Themes: Benefits of sex work included higher income and independent working schedule. Risks include dangerous work environment, including violence (physical and sexual) from clients and police. Sex workers regularly faced social discrimination from the public, service providers and their families. Poverty increased HIV risk and sex workers accepted more money for unprotected sex. |
| Mbonye, 2012 | Kampala | Qualitative: life histories | To explore sex workers lives from childhood to adult life and identify factors that enabled entry into sex work | N=58 Ugandan female sex workers | | Themes: Many sex workers experienced childhood adversity including neglect and abuse from parents/teachers. Early unwanted pregnancy led many to leave school. Common reason for starting sex work was the support children and pay school fees. Violence from clients and police was common. | | --- | |
| Mbonye, 2013 | Kampala | Qualitative: 3 in-depth interviews per participant | To explore key risk factors associated with different sex work environments. | N=58 Ugandan female sex workers | Themes: Common sex work solicitation venues included street/roadsides, bars and nightclubs. Common service venues included lodges, bars, dark alleyways or parking lots. Outdoor locations were more dangerous – increased police harassment, client violence, and stigma from public. |
| Scorgie, 2013 | Multisite: Uganda (Kampala), Kenya, South Africa, Zimbabwe | Qualitative: 55 in-depth interviews, 12 focus groups | To explore the impact of violence and related human rights abuses among sex workers | N= 136 sex workers, female (n=106), male (n=26) and transgendered (n=4);  n=25 sex workers from Uganda | Themes: Physical/sexual violence and condom refusal from clients was common. Police harassment including physical/sexual assault and extortion was common and legitimized through criminalization. In addition to police, other authorities, landlords and brothel owners demanded sex in exchange for safety, freedom and resources. |
| Scorgie, 2013 | Multisite: Uganda (Kampala), Kenya, South Africa, Zimbabwe | Qualitative: 55 in-depth interviews, 12 focus groups | To explore barriers to care among sex workers in public and private clinics, and identify strategies to improve service provision. | N= 136 sex workers, female (n=106), male (n=26) and transgendered (n=4);  n=25 sex workers from Uganda | Key unmet health needs included diagnosis and treatment for sexually transmitted infections and insufficient access to condoms and lubricant. Care providers often denied services and treatment following physical/sexual assault. Sex workers reported that private clinics were more welcoming, had higher quality of services and more respect for dignity and confidentiality compared to public clinics. Sex workers called for the decriminalization of sex work and more community-based programming. |
| Nyanzi, 2013 | Kampala and IDP camps in northern Uganda | Qualitative: ethnographic, participant observation, repeat in-depth interviews, focus group, policy review, media analysis | To explore the experiences of male, female and transgendered refugees who are engaged in sex workers | N=54 male, female, transgendered and queer refugees involved in sex work | Themes: Violence from clients was very common, including homophobic rape, gang rape, and being drugged. Policing was an extra concern among refugees because of statelessness. Poverty was driving unprotected sex. Criminalization of sex work and homosexuality legally limited access to care from all sources (UN, government, NGO). |
| Mbonye, 2014 | Kampala | Qualitative: 3 in-depth interviews per participant | To assess the magnitude, driving factors and consequences associated with alcohol consumption among sex workers | N=40 Ugandan female sex workers | Themes: Many began drinking as an emotional coping strategy to gain courage to engage in sex work. Consequences of alcohol use included difficulty negotiating condom use, clients buying sex workers drinks in place of paying for sex, and intoxicated clients were more violent. |
| Marlow, 2014 | Kampala | Qualitative: in-depth interviews | To investigate sex workers experiences with post-abortion care | N=9 Ugandan female sex workers who just had an abortion | Themes: Most common reasons for abortion included: not knowing the father, could not afford another child, and the child was the result of a rape. Stigma against sex workers and the stigma of having an abortion were dual barrier to care. |
| Lees, 2014 | Multisite: Uganda (Kampala)/Tanzania | Qualitative: semi-structured interviews | To investigate the motivations for intervaginal practices | N= 176 female sex workers,  n=96 from Uganda | Themes: intervaginal practices (douching, cleansing with soap/water, inserting herbs) were motivated by overlapping concerns with hygiene, morality, sexual pleasure, fertility, relationship security, and economic security. |
